# Clinical efficacy of modified suanzaoren decoction compared to esazolam tablets in the treatment of chronic insomnia disorder

**DOI:** 10.3389/fpsyt.2025.1533652

**Published:** 2025-07-24

**Authors:** Ping Yao, Xingyan Guo, Zhiguo Guo, Wuhong Lin, Min Liu, Min Chen, Jie Li, Long-Biao Cui, Dongsheng Lv

**Affiliations:** ^1^ Inner Mongolia Mental Health Center (The Third Hospital of Inner Mongolia Autonomous Region, Brain Hospital of Inner Mongolia Autonomous Region), Hohhot, China; ^2^ Department of Radiology, The Second Affiliated Hospital of Xi’an Jiaotong University, Xi’an, China; ^3^ Shaanxi Provincial Key Laboratory of Clinic Genetics, Fourth Military Medical University, Xi’an, China; ^4^ Department of Psychiatry, Xijing 986 Hospital, Fourth Military Medical University, Xi’an, China

**Keywords:** insomnia disorder, Suanzaoren decoction, estazolam tablets, clinical efficacy, cognitive function

## Abstract

**Background:**

Traditional Chinese medicine is one of the important methods for treating chronic insomnia disorder (CID).

**Aims:**

We aimed to observe the multi-dimensional clinical outcomes of modified suanzaoren decoction (SZRD) compared to esazolam tablets in the treatment of CID patients.

**Methods:**

A total of 80 patients with CID were divided into two treatment groups, and were given modified SZRD and esazolam tablets treatment respectively for 6 weeks. The Pittsburgh Sleep Quality Index (PSQI), Insomnia Severity Index, Hamilton Anxiety Scale, Hamilton Depression Scale, polysomnography (PSG), repeated battery for the assessment of neuropsychological status were performed to assess the changes of subjective and objective sleep, mood, and cognitive function.

**Results:**

Intra-group improvement: Compared to before treatment, both the modified SZRD and estazolam groups showed improvements in subjective sleep, depression, anxiety, immediate memory, and delayed memory scores (*P*<0.05). Inter-group comparison: There was a significant difference between the modified SZRD and estazolam groups in subjective PSQI scores (*P*=0.033). Based on PSG objective assessment results, both the estazolam and modified SZRD groups demonstrated a significant increase in N3 stage sleep (slow-wave sleep) duration compared to before treatment (*P*=0.037). However, there was no statistically significant difference in the effect size between the two groups (*P*>0.05), indicating that both interventions were equivalent in improving deep sleep. Nevertheless, residual variance analysis indicates that estazolam showed enhanced predictive stability in subjective sleep quality assessed by ISI (SSR=11.73 vs. 31.19; *F*=13.39, *P*<0.001), while modified SZRD exhibited enhanced predictive stability in objective slow-wave sleep maintenance, specifically in N3 stage duration (SSR=703.11 vs. 1761.08; *F*=4.98, *P*=0.029).

**Conclusion:**

After the treatment of CID with the modified SZRD and esazolam, they have the comparable clinical efficacy. However, estazolam showed a more consistent treatment effect in subjective sleep quality assessment among the study population, whereas modified SZRD showed a more consistent treatment effect in objective slow-wave sleep maintenance, specifically in N3 stage duration. The registration number was NCT06452953.

## 1 Introduction

The global prevalence of insomnia symptoms is approximately 30% to 35%, and the prevalence of insomnia in China is about 15%. According to the diagnostic criteria such as those defined by the Diagnostic and Statistical Manual of Mental Disorders (DSM) and the International Classification of Sleep Disorders (ICSD), the prevalence rate of insomnia disorder ranges from 10% to 20% (1, 2). Chronic insomnia disorder affects approximately 10% of the European population, and this proportion may be even higher in Italy (3). The prevalence of insomnia in the general population of Saudi Arabia is as high as 37.6% (4). Research has indicated that large variations in the prevalence of insomnia depending on the region of the world, but always with a significant variation between sites in the same region (5). Therefore, chronic insomnia disorder has become a widely recognized social and public health issue. Chronic insomnia disorder (CID) is defined as experiencing insomnia symptoms and daytime functional impairments at least three times a week for at least three months (6, 7). Currently, there are many methods to treat CID, including non-pharmacological treatments such as cognitive behavioral therapy for insomnia (CBT-I) (8, 9). However, CBT-I often requires therapists with professional knowledge, and has a slow onset of effect, poor patient adherence, and a high dropout rate, requiring therapists to invest a lot of time and energy which limits its clinical application, and thus its use is still not widespread. Transcranial magnetic stimulation (TMS) treatment is limited by treatment time, high costs, and difficulty in clinical promotion, so drug therapy is still commonly used in clinical practice. Drug treatments usually include benzodiazepines and non-benzodiazepines, melatonin receptor agonists, orexin receptor antagonists, and antidepressants. Clinically, benzodiazepines are the most widely used, among which estazolam is a commonly used benzodiazepine drug. However, these drugs often have significant next-day residual effects, affecting daytime activities, and can easily lead to tolerance and dependence issues (10). After discontinuation, rebound insomnia is likely to occur, potentially impairing cognitive function. Although they have significant short-term efficacy, the long-term effectiveness diminishes, and they come with many adverse reactions, making the treatment outcomes less satisfactory.

In contrast, traditional Chinese medicine (TCM) treatment methods have a calming and sleep-inducing effect, with fewer side effects and higher compliance, making them one of the important methods for treating CID. TCM is a main part of complementary and alternative therapies (11), includes Chinese herbal medicine (CHM), acupuncture, massage (12). CHM formulae are a combination of several CHMs according to TCM theory Jun-Chen-Zuo-Shi, first recorded by Huangdi Neijing (Inner Canon of the Yellow Emperor) (13). Suanzaoren Decoction (SZRD) is a famous TCM formula for treating insomnia due to yin-deficiency and fire-excess syndrome. It can nourish yin and has a moistening effect, thus eliminating excess heat, relieving irritability, and regulating liver blood while dredging the liver’s Qi (14, 15). Suanzaoren was first recorded in the ShennongBencao Jing (Shennong’s Classic of Materia Medica), the earliest medicine monograph of China written 2500 years ago (16). It is composed of five medicinal herbs: *Ziziphus jujuba* Mill (Suanzaoren), *Glycyrrhiza uralensis* Fisch (Licorice), *Anemarrhena asphodeloides* Bunge (Zhimu), *Poria cocos* (Fuling), and *Ligusticum chuanxiong* (Chuanxiong) (17). Clinical studies have shown that SZRD can improve subjective sleep quality and has good safety and tolerability (14, 18, 19). In traditional Chinese medicine clinical practice, based on the principle of syndrome differentiation and treatment, it is common to modify the traditional SZRD by adding or subtracting other herbs to form a modified formula, aimed at enhancing its sedative and sleep-promoting effects, with Suanzaoren being a required ingredient. Treatment with modified SZRD in CID patients can improve cognitive function, with cognitive scores positively correlated with total sleep time, deep sleep time, and REM sleep time, and negatively correlated with wake time, sleep onset latency, and light sleep time. This suggests that changes in cognitive function are related to sleep duration, sleep continuity, and sleep architecture, possibly due to increased deep sleep time and improved sleep structure (20). These findings suggest that SZRD may be an effective method for treating insomnia. However, current evaluations of its treatment efficacy are mostly based on clinical symptom scales, which are subjective, and there are few objective assessment indicators like cognitive function.

In summary, this study aims to treat patients with Chronic Insomnia Disorder (CID) using modified SZRD, combined with overnight polysomnography and cognitive assessment, to analyze the effects of the modified SZRD on sleep and cognition. The goal is to explore the clinical efficacy for patients, provide a reference for treating CID, and assist in clinical diagnosis and targeted therapy.

## 2 Methods

### 2.1 Subjects recruitment

This study adopts a prospective, open-label, case-control study design. Patients were recruited from the outpatient department of the Inner Mongolia Mental Health Center and from the community between October 1, 2020, and September 1, 2023. The diagnostic criteria were based on the International Classification of Sleep Disorders, 3rd Edition (ICSD-3) (21), and diagnoses were made by professional psychiatrists. The diagnosis required the concurrent presence of yin-deficiency and fire-excess syndrome, as evaluated by a traditional Chinese medicine practitioner. Participants were aged 18–65 years, had at least 9 years of education, and were right-handed. A total of 95 CID patients were initially enrolled in the study. Among them, 3 were excluded due to polysomnography results indicating severe sleep apnea syndrome, 5 were excluded due to time constraints or unwillingness to continue medication, and 7 were unable to continue with polysomnography. Ultimately, 80 CID patients remained and were divided into two groups for treatment, receiving modified SZRD and estazolam tablets, respectively, for a 6-week period. Estazolam is a frequently prescribed benzodiazepine medication, widely prescribed in clinical practice for the treatment of insomnia due to its potent hypnotic and anxiolytic effects, as well as its relatively high intermediate-to-long duration of action. Moreover, both estazolam and the modified SZRD may exert their sedative and hypnotic effects by modulating the levels of the neurotransmitter γ-aminobutyric acid (GABA) in the brain (11, 22). In accordance with medical ethics requirements, all patients signed informed consent forms, adhering to the principles of informed consent and confidentiality. The research was approved by the Ethics Committee of the Inner Mongolia Mental Health Center, and that informed consent was obtained.

Exclusion Criteria:

- Use of sedative-hypnotic drugs or psychiatric medications within one week prior to enrollment;
- Habitual consumption of strong tea, heavy smoking, or caffeine dependence;
- Women who are in the pre-pregnancy period or breastfeeding;
- Patients with severe cardiovascular and cerebrovascular diseases, other psychiatric disorders, or severe obstructive sleep apnea syndrome;
- Shift workers;
- Individuals with allergic constitutions or multiple drug allergies;
- Patients who are allergic to the main ingredients or excipients, or have drug contraindications.

Criteria for Dropout, Removal, and Discontinuation:

- Patients who develop primary physical diseases or psychiatric disorders during the treatment;
- Patients who develop severe complications making them unsuitable to continue with the study treatment;
- Patients who are unable to continue the treatment for other reasons, or who voluntarily withdraw;
- Patients with poor compliance.

### 2.2 Intervention methods

Traditional Chinese Medicine Group: The modified SZRD, primarily composed of *Suanzaoren (Ziziphus jujuba* Mill*)* 30.0g*, Chuanxiong (Ligusticum chuanxiong* S.H.Qiu, Y.Q.Zeng, K.Y.Pan, Y.C.Tang & J.M.Xu*), Zhimu (Anemarrhena asphodeloides* Bunge*)*, and *Fuling (Poria cocos)* 15.0g each*, Licorice (Glycyrrhiza uralensis* Fisch*)* 3.0g*, and Amber (Succinum)* 3.0g. The Chinese herbal granules for SZRD and Amber powder were provided by Sichuan Xinyisheng Pharmaceutical Co., Ltd., batch number: 2107020. The decoction was taken with warm water 30 minutes after breakfast and dinner.

Western Medicine Group: Estazolam tablets (Shandong Xinyi Pharmaceutical Co., Ltd., [Approval Number] National Drug Approval No. H370230047, specification: 1mg/tablet), one tablet taken before bedtime. During the treatment, no other sedative or hypnotic drugs were used. The treatment course lasted for 6 weeks.

### 2.3 Clinical evaluation

(1) Pittsburgh Sleep Quality Index (PSQI) (23): PSQI scores were assessed for both groups before treatment and one month after treatment, based on the indicators of each dimension of the scale. Efficacy evaluation: a reduction rate of PSQI ≥75% was considered cured, 50%-74% was significantly effective, 25%-49% was effective, and<25% was ineffective (24). The total effective rate of the drug was calculated as (cured + significantly effective + effective)/total number of cases × 100%.

1. Repeatable Battery for the Assessment of Neuropsychological Status (RBANS): Cognitive function of patients was assessed before and after treatment (25).
2. Objective Sleep Evaluation Indicators:

Overnight polysomnography (PSG): Overnight PSG monitoring was conducted by professional technical personnel in the sleep monitoring ward of the Sleep Medicine Center at the Inner Mongolia Mental Health Center, and sleep stages and related events were reported according to the standards of the American Academy of Sleep Medicine (26), including non-rapid eye movement sleep (NREM) stages N1, N2, N3, and rapid eye movement (REM) sleep.

The monitor should be aware of the following adaptation night protocol when scheduling a PSG: a. No smoking or drinking alcohol; b. To ensure signal acquisition during monitoring, turn off your phone for the entire night; c. Patients who sweat easily or are afraid of heat should inform the staff in advance to adjust the room temperature; d. During the examination period, only bedside toilets can be used. Patients with nocturnal habits should place their chamber pot and slippers on the same side as the monitor for easy access; e. Do not read books, newspapers, or use mobile phones after the test starts; f. If necessary or uncomfortable at night, please press the bedside call button to notify the on-duty personnel; g. Please relax and fall asleep during the monitoring process; you can change positions freely to make yourself comfortable; h. The staff will stay overnight. In case of electrode or abnormal monitoring signals during the monitoring process, the staff will handle it, no need to worry; i. The test ends around 6 a.m., and medical staff will remove the electrodes for you. Please wait patiently. Do not remove the electrodes yourself to avoid damaging them, as they are expensive and may require compensation if damaged; j. After removing the electrodes, please wait for the nurse to measure your blood pressure before getting up and moving around; k. To ensure monitoring quality, accompanying persons should not sleep in the same room as patients. They can place the accompanying bed outside the ward in advance. Accompanying persons cannot enter the monitoring room at will during the monitoring period; l. After finishing the morning monitoring, patients undergoing MSLT/MWT should finish eating by 7:30 a.m. Breakfast should be light and not too full, avoiding foods and beverages containing caffeine (such as coffee, chocolate, or tea). Stay awake until daytime monitoring begins. If the patient is extremely sleepy, please communicate with the night shift technician in advance to prevent difficulty falling asleep and ensure accurate monitoring; m. Private items, especially water and drinks, must not be placed on the monitoring instrument cabinet.

The duration of a PSG measurement typically spans from 10 PM to 6 AM. Participants are instructed to maintain a fixed sleep schedule (23:00-7:00) for 3 days prior to the test. On the testing day: a. 19:00: Fasting begins (except for small amounts of water); b. 20:00: Arrival at the lab, completion of informed consent; c. 21:00: Electrode application and calibration (10–20 system, impedance<5 kΩ); d. 22:00: Lights out (monitoring begins); e. 06:00: Lights on (monitoring ends).

The PSG scoring and interpretation were performed in accordance with the American Academy of Sleep Medicine (AASM) Manual Version 2.6 scoring criteria. Data Quality Control: Ensure≥6 hours of usable sleep data, otherwise, a repeat test is required. The PSG results were jointly scored by two China-certified sleep technologists, with any discrepancies resolved by a third senior technologist.

3. Insomnia Severity Index (ISI): The severity of insomnia was assessed using the ISI (27).
4. Epworth Sleepiness Scale (ESS): Daytime sleepiness was assessed using the ESS (27).
5. The Hamilton Depression Scale (HAMD) and the Hamilton Anxiety Scale (HAMA) were used to assess patients’ depression and anxiety levels.

To ensure the accuracy and reliability of data collection, each patient participating in this study was evaluated by at least two psychiatrists, and uniform training was provided in terms of operational procedures and guidelines.

### 2.4 Statistical analysis

Statistical analysis of clinical characteristics was conducted using SPSS 26.0 and R4.4.3 software. Data conforming to a normal distribution were analyzed using the independent samples *t*-test. Data not conforming to a normal distribution were analyzed using the rank-sum test. Categorical data were analyzed using the chi-square test, with a significance level of α=0.05. The linear mixed-effects model (LME) analyses were performed to investigate the effects of demographic variables and intervention conditions on scores for each psychological scale. For each scale, the full model included fixed effects of age (in years, continuous), education level (Edu, in years, continuous), Gender (binary), intervention group (binary, estazolam vs. SZRD), time point (Time, binary, baseline vs. follow-up), and their interaction (Group×Time). Random intercepts for participants were included to account for individual heterogeneity. The model formula was specified as:


$$y_{ij}=\beta_{0}+\sum_{k=1}^{3}\beta_{k}x_{ik}+\beta_{4}{ɡ}_{i}+\beta_{5}t_{ij}+\beta_{6}\left({ɡ}_{i}\times t_{ij}\right)+u_{i}+\in_{ij},$$

Where $y_{ij}$ is the outcome score for the i-th participant at the j-th measurement time point, $\beta_{0}$ is the global intercept, $\beta_{1}-\beta_{6}$ are the fixed-effect coefficients, $u_{i}\sim N\left(0,\;\sigma^{2}\right)$ is the random intercept for i-th participant, and $\in_{ij}$ is the residual which is different across every combination of Group and Time. The Akaike Information Criterion (AIC) value was used to select models: Every subset of the covariates (age, Edu, and gender) was evaluated, and then the model with the minimum AIC value was chosen as the optimal one.

The predictive stability of the intervention methods was assessed by comparing residual variances from linear regression models. For each psychological scale, separate linear regression models were fitted to the estazolam and SZRD groups, using the covariate subsets (age, Edu, and gender) previously selected in the LME analysis. Residual variances between the two groups were formally compared using Levene’s test for equality of variances. Lower residual variance in a group indicates higher predictive precision, reflecting greater stability of the corresponding intervention method.

## 3 Results

### 3.1 Demographic and clinical characteristics

A total of 80 patients were finally recruited for this study and were divided into two groups to complete treatment with either modified SZRD (51 cases) or estazolam (29 cases). There were no statistically significant differences between the two groups in terms of gender (*P*=0.104), age (*P*=0.929), years of education (*P*=0.943), and duration of illness (*P*=0.864). See **Table 1** for details.

**Table 1:** Subject demographic and clinical information.

Clinical characteristics	SZRD group n=51	Estazolam group n=29	*Z/χ^2^*	*P*
Gender (Male/Female)	13/38	3/26	2.65^a^	0.104
Age (Years)	45.90 ± 11.95	46.14 ± 10.32	-0.09^b^	0.929
Education duration (Years)	15.00 (12.00,16.00)	15.00 (12.00,16.00)	-0.07^c^	0.943
Disease duration (Years)	5.00 (2.00,10.00)	4.00 (2.50,10.00)	-0.17^c^	0.864

SZRD group, Suanzaoren Decoction treatment group; Estazolam group, estazolam treatment group; a, Chi-square test was used; b, Independent samples t-test was used; c, Wilcoxon rank-sum was used. Data are shown as Mean ± SD (normally distributed) or Median (Q1, Q3) (non-normally distributed).

#### 3.1.1 Comparative analysis of primary clinical evaluation data across modified SZRD and estazolam group

The effective rate of the modified SZRD group was 78.40%, and the effective rate of the estazolam group was 72.40%, indicating that the modified SZRD also has comparable efficacy relative to estazolam in the treatment of insomnia disorder (**Table 2**). The results of the general linear model revealed that after treatment with both medications, there were significant time effects on the PSQI (*P*<0.001), ISI (*P*<0.001), total scores of RBANS (*P*=0.009), immediate memory scores of RBANS (*P*=0.013), and delayed memory scores of RBANS (*P*<0.001) in patients with insomnia, suggesting that both modified SZRD and estazolam can effectively improve patients’ sleep quality, immediate memory and delayed memory function. However, there were no statistically significant improvements in other cognitive function indicators for either treatment (*P*>0.05). Additionally, the group effect demonstrated that there was a significant difference in PSQI scores between the groups (*P*=0.030), indicating a significant difference in sleep quality improvement between the two groups, as shown in **Table 3**.

**Table 2:** Comparative analysis of clinical efficacy between modified SZRD and estazolam group.

Item	Name	Groups		Total	*χ^2^*	*P*
		SZRD group	Estazolam group			
Efficacy	Effective rate (%)	40 (78.40)	21 (72.40)	61 (76.20)	0.37	0.543
	Ineffective rate (%)	11 (21.60)	8 (27.60)	19 (23.80)		
Total		51	29	80		

SZRD group, Suanzaoren Decoction treatment group; Estazolam group, estazolam treatment group.

**Table 3:** Comparative analysis of primary clinical evaluation data across the two groups before and after treatment.

Variables	Time		Group		Time×Group	
	Estimate (95% CI)	*P*	Estimate (95% CI)	*P*	Estimate (95% CI)	*P*
PSQI	-5.68 (-6.64, -4.73)	<0.001	-1.32 (-2.51, -0.13)	0.033	0.07 (-1.25, 1.39)	0.918
ISI	-12.60 (-13.95, -11.26)	<0.001	-2.37 (-4.76, 0.01)	0.055	1.68 (-0.41, 3.77)	0.119
RBANS						
Total scores	5.52 (1.51, 9.52)	0.009	0.07 (-4.37, 4.501)	0.976	2.24 (-3.37, 7.845)	0.437
Immediate memory scores	7.99 (1.87, 14.10)	0.013	-3.62 (-12.66, 5.42)	0.435	5.18 (-2.28, 12.64)	0.178
Visual span scores	0.76 (-2.29, 3.81)	0.628	0.00 (-3.79, 3.79)	0.999	-0.84 (-7.14, 5.46)	0.795
Language function scores	2.03 (-1.56, 5.62)	0.272	0.73 (-4.15, 5.62)	0.770	2.36 (-3.99, 8.72)	0.469
Attention scores	2.12 (-3.76, 8.01)	0.482	1.57 (-5.48, 8.61)	0.665	0.61 (-7.46, 8.68)	0.882
Delayed memory scores	9.13 (3.84, 14.41)	0.001	1.82 (-2.66, 6.31)	0.430	-0.96 (-8.63, 6.70)	0.807

CI, Confidence Intervals; PSQI, Pittsburgh Sleep Quality Index; ISI, Insomnia Severity Index; RBANS, Repeatable Battery for the Assessment of Neuropsychological Status.

#### 3.1.2 Comparative analysis of secondary clinical evaluation data

The analysis of secondary clinical data indicates that after the intervention with both drugs, significant time effects were observed in the assessments of HAMD (*P*<0.001), HAMA (*P*<0.001) and ESS (*P*=0.031) scale scores, while group effects and interaction effects did not reach statistical significance (*P*>0.05). This demonstrates that both estazolam and modified SZRD have exerted significant therapeutic effects in alleviating patients’ anxiety, depression, and excessive daytime sleepiness symptoms, with no significant difference in efficacy between the two treatments, as shown in **Table 4**.

**Table 4:** Comparison of secondary clinical variables across the two groups before and after treatment.

Variables	Time		Group		Time×Group	
	Estimate (95% CI)	*P*	Estimate (95% CI)	*P*	Estimate (95% CI)	*P*
HAMD	-5.16(-6.71, -3.60)	<0.001	0.01 (-2.34, 2.37)	0.992	-0.25(-2.11, 1.61)	0.791
HAMA	-5.22(-6.53, -3.92)	<0.001	-0.91 (-2.89, 1.06)	0.366	0.73 (-0.85, 2.31)	0.367
ESS	-1.37 (-2.59, -0.14)	0.031	0.09 (-1.70, 1.87)	0.925	0.67 (-0.87, 2.22)	0.386

CI, Confidence Intervals; HAMD, Hamilton Depression Scale; HAMA, Hamilton Anxiety Scale; ESS, Epworth Sleepiness Scale.

### 3.2 Results of objective PSG parameters

Objective PSG data reveal that following the intervention with both drugs, there were no significant changes in total sleep time, sleep efficiency, sleep latency, REM sleep latency, REM sleep duration, and the duration of N1 and N2 stages in terms of time effects, group effects, and interaction effects, indicating that these two drugs were not significantly effective in improving the aforementioned objective sleep parameters (*P*>0.05). However, it is noteworthy that a significant time effect was observed for the duration of N3 sleep (*P*=0.031), while the group effect and interaction effect did not reach significance levels, suggesting that both estazolam and modified SZRD exerted significant therapeutic effects in enhancing deep sleep, with no significant difference in efficacy between the two treatments, as shown in **Table 5**.

**Table 5:** The comparison of PSG across the two groups before and after treatment. .

Variables	Time		Group		Time×Group	
	Estimate (95% CI)	*P*	Estimate (95% CI)	*P*	Estimate (95% CI)	*P*
PSG						
Total sleep time (min)	13.56 (-10.81, 37.93)	0.281	-5.85 (-34.61, 22.91)	0.692	69.13 (-62.32, 200.58)	0.308
Sleep efficiency (%)	0.196 (-4.93, 5.32)	0.941	-2.92 (-9.42, 3.59)	0.382	5.51 (-1.88, 12.91)	0.148
Sleep latency (min)	11.76 (-0.72, 24.23)	0.068	-1.77 (-10.05, 6.50)	0.675	-10.52 (-25.50, 4.45)	0.171
REM latency (min)	-19.94 (-43.40, 3.53)	0.100	-3.98 (-54.78, 46.81)	0.878	6.88 (-43.66, 57.41)	0.786
REM sleep (min)	6.34 (-6.19, 18.86)	0.325	-9.33 (-22.80, 4.14)	0.179	5.17 (-10.47, 20.81)	0.519
N1 (min)	-9.65 (-19.83, 0.53)	0.067	-1.50 (-15.61, 12.62)	0.836	3.27 (-9.25, 15.79)	0.610
N2 (min)	-4.07 (-26.03, 17.88)	0.716	-3.80 (-27.61, 20.01)	0.755	14.45 (-12.63, 41.53)	0.298
N3 (min)	17.54 (1.38, 33.72)	0.037	2.89 (-6.56, 12.34)	0.550	-8.49 (-26.43, 9.45)	0.357

CI, Confidence Intervals; PSG, Polysomnography; REM, Rapid Eye Movement Stage; N1, Non-REM Sleep Stage 1; N2, Non-REM Sleep Stage 2; N3, Non-REM Sleep Stage 3.

### 3.3 Evaluation of intervention effect robustness based on heterogeneity of residual variances: comparison of clinical prediction stability between modified SZRD and estazolam group

To evaluate the differential predictability of estazolam and SZRD across clinical indicators, we compared their residual sum of squares (SSR) through variance homogeneity testing. As shown in **Table 6**, estazolam demonstrated superior predictive stability for ISI (SSR=11.73vs.31.19; *F*=13.39, *P*<0.001). In contrast, SZRD showed a unique advantage in predicting N3 sleep stage maintenance (SSR=703.11 vs. 1761.08; *F*=4.98, *P*=0.029), with its lower SSR highlighting greater reliability in modeling slow-wave sleep dynamics. All comparisons reached statistical significance (*P*<0.05), confirming the intervention-specific predictability patterns.

**Table 6:** Comparison of predictability of two groups of drugs for clinical indicators - based on variance test results of data residual sum of squares.

Item	Groups (SSR)		F	P
	Estazolam	SZRD		
ISI	11.73^a^	31.19	13.39	<0.001
N3 (min)	1761.08	703.11^a^	4.98	0.029

SSR, Sum of Squared Residuals; ISI, Insomnia Severity Index; N3, non-REM sleep stage 3; A smaller SSR indicates a better model fit; a: higher level of predictive stability.

## 4 Discussion

In recent years, the treatment methods of chronic insomnia have become more and more abundant. Traditional Chinese medicine has received extensive attention because it can effectively improve the sleep status of patients with chronic insomnia and avoid drug abuse and dependence. The advantages of various clinical treatment methods need to be further explored. This study investigated the clinical efficacy and cognitive function changes before and after the treatment of CID by modified SZRD and estazolam. The research protocol excluded the influence of secondary sleep disorders and mental disorders, and evaluated the sleep effect by subjective and objective indicators. The results showed that the therapeutic efficacy of the two groups was similar. The effective rate of modified SZRD was 78.4%, and the effective rate of estazolam was 72.4%. After treatment, the clinical symptoms of CID in both groups were improved, sleep quality was enhanced, and some cognitive functions were also improved. In addition, the HAMD and HAMA scores of CID patients in both groups were lower than those before treatment, with no significant difference between the two groups, indicating that modified SZRD can not only improve insomnia symptoms but also reduce anxiety and depression like western medicine. It is noteworthy that estazolam exhibited a more consistent therapeutic effect on subjective sleep quality assessment among the study population, while SZRD demonstrated enhanced predictive stability in the objective maintenance of slow-wave sleep (duration of N3 stage).

Wu Wangfang and other research results also show that (20, 28), the treatment of modified SZRD has obvious curative effect on CID patients, can improve subjective sleep quality, the effect is comparable to that of Western medicine, and there are no obvious side effects during the medication process; another study also found that the use of modified SZRD in the treatment of insomnia patients has obvious effect, can effectively improve the treatment satisfaction of patients, improve the patient’s anxiety, depression scale scores and PSQI scores, proving that modified SZRD can effectively reduce anxiety and depression in the treatment of CID patients, and the most important thing is to improve insomnia symptoms, and the safety is high (29). Another study showed that modified jujube seed decoction can reduce the severity of insomnia in patients, reduce the level of past arousal, and improve the clinical severity of patients with anxiety insomnia. It is an effective intervention for the treatment of chronic insomnia (30). The above results are similar to this study. The suanzaoren typically contains complex mixtures of phytochemicals including sanjoinine A, Jujuboside A, spinosin and other flavonoids, which has sedative and hypnotic functions primarily mediated by the GABAergic and serotonergic system (11). Jujube seed decoction may also play a sedative and hypnotic role in anti-anxiety and depression by regulating the levels of neurotransmitters and signaling pathways such as serotonin and norepinephrine (31, 32).

Estazolam, as a classic benzodiazepine, has been used in the management of insomnia for several decades. Estazolam may enhance sleep by potentiating GABA binding to GABAa receptors and elevating GABA levels, thereby inhibiting signal transduction in wakefulness-promoting neuronal circuits, ultimately reducing sleep latency and extending sleep duration (22). As a primary inhibitory neurotransmitter, GABA plays a key role in regulating sleep-wake cycles and maintaining the overall balance between neuronal excitation and inhibition within the central nervous system (33, 34). Therefore, estazolam and SZRD may both exert their sedative and hypnotic effects by regulating the levels of the GABA in the brain.

Previous studies have used different cognitive measures to conduct neuropsychological studies on CID patients, and all found that CID affects cognitive function (35). This study found that after drug treatment, the total scores, delayed memory and immediate memory in the RBANS cognitive assessment of the two groups of CID patients were significantly improved. Although there was no significant difference in other cognitive functions between the two groups before and after treatment, a noticeable difference was observed in delayed memory, indicating that modified SZRD also has an advantage in improving certain cognitive functions. The Fortier-Brochu (36) study compared the CID patients with healthy people and found that there was no significant difference in alertness, orientation, and execution in the attention network between the two groups. Perrier J (37) believes that patients with long-term insomnia often show damage to the attention network, and the research results are not consistent. Our study also found that there is no improvement in cognitive attention in CID patients after drug treatment, so whether chronic insomnia affects attention needs to be further explored. This study found that after treatment with modified SZRD, the delayed memory in CID patients were significantly improved compared with those before treatment, indicating that modified SZRD has a certain improvement in cognitive function in CID patients, mainly in the improvement of memory, which is consistent with Zhang Yonghua (20, 28) research. Modified SZRD improve subjective and objective sleep and cognitive function in CID patients. These studies also show that chronic insomnia may change people’s cognitive function to a certain extent, and after drug treatment, sleep quality and cognitive function can be improved. However, the results of this study may be limited by a single dose and a shorter medication time. If there is a longer treatment time and follow-up, it may better reflect the improvement of modified jujube seed decoction on sleep and cognition. At the same time, no abnormalities were found in the hematuria test and electrocardiogram of the enrolled patients. Previous studies have shown that modified jujube seed decoction has good safety and tolerability, which is consistent with the conclusion of a recent review (13).

Through in-depth analysis of intervention-specific predictive patterns, we have identified significant differences between estazolam and SZRD in predicting the efficacy across various clinical indicators. This discovery not only highlights the unique characteristics of each medication but also provides clinicians with solid and important grounds for drug selection. Specifically, the stable predictability of estazolam in assessing insomnia severity and the notable advantage of SZRD in maintaining the N3 sleep stage offer robust data support for the implementation of personalized treatment strategies. Moving forward, we anticipate further exploring the predictive potential of these drugs on other clinical indicators, while also delving into their underlying mechanisms of action, with the aim of offering more comprehensive and precise treatment guidance to clinicians.

This study has the following shortcomings: first, this topic did not collect the longitudinal health control group before and after, and did not conduct matching health control analysis; secondly, in the future, a large sample database can be constructed to strengthen case follow-up tracking, and provide a certain scientific basis for the treatment and remission of CID patients in the future. Thirdly, considering that altering estazolam to a decoction might affect its efficacy and that converting traditional herbal decoctions into tablets presents significant challenges, we did not employ a blind method in this study. This could result in a psychological placebo effect among patients who have faith in TCM. In future related research, we plan to prepare both medications in a similar powdered form to conduct a blind study, thereby eliminating potential biases that may arise from the absence of blinding. Lastly, we did not conduct a detailed study on side effects. In our subsequent research, we hope to explore whether different medications lead to different side effects.

In summary, compared with estazolam, modified SZRD can not only improve the subjective sleep quality of patients with CID, but also improve the cognitive function of patients. Modified SZRD has better effectiveness in the treatment of CID, and the efficacy of estazolam is comparable. It overcomes the shortcomings of some previous sedative and hypnotic drug treatments.

This study investigated the clinical efficacy and cognitive function changes before and after the treatment of CID with modified SZRD and estazolam, and found that the clinical symptoms, mood, and cognitive function (especially immediate memory and delayed memory) of CID patients improved after drug treatment. However, compared with estazolam treatment, modified SZRD was more effective in improving subjective sleep quality, and the language function, REM duration and N3 duration of patients treated with modified SZRD increased significantly.

## Data Availability

The raw data supporting the conclusions of this article will be made available by the authors, without undue reservation.
