# Supplementary figures and images for: Clinical efficacy of modified suanzaoren decoction compared to esazolam tablets in the treatment of chronic insomnia disorder
Source: Front Psychiatry. 2025 Jul 24;16:1533652. doi: 10.3389/fpsyt.2025.1533652 (PMC12328296; doi:10.3389/fpsyt.2025.1533652)

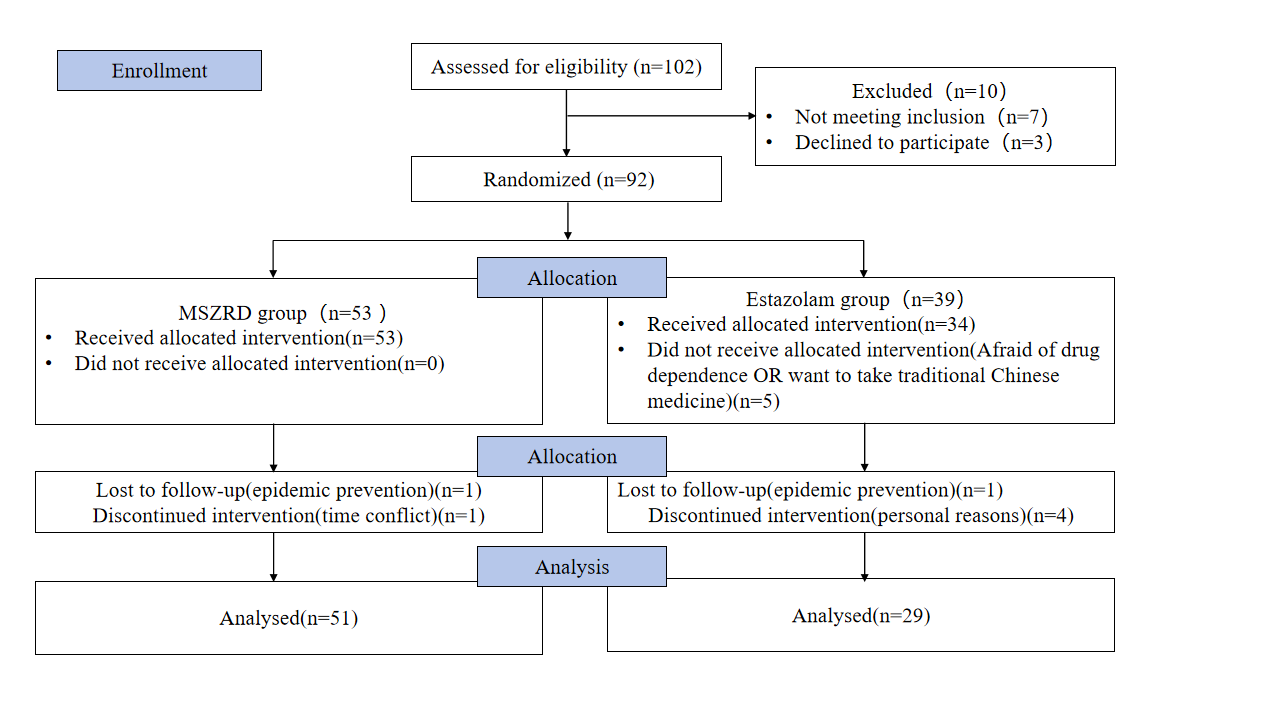

Supplement: Supplementary file 1 [file Image1.tif]
